# Integrating genetics in the care of children born with cleft lip and palate^☆^

**DOI:** 10.1016/j.jped.2024.08.006

**Published:** 2024-09-04

**Authors:** Jonathan R. Sandy

**Affiliations:** University of Bristol, Bristol, United Kingdom

Children born with cleft lip and palate (CLP) require care from a wide range of healthcare disciplines and they can have a difficult and unhappy life, particularly in the early years. Clefts are the most common craniofacial anomaly in humans and phenotypic expression is variable. Clefts of the palate alone are the most common comprising typically 50 % of all clefts. Unilateral clefts of the lip and palate are seen in about 25 % of these children, bilateral cleft lip and palate (10 %), and cleft lip alone (10 %). Rarer phenotypic expressions account for the remainder. The cause of cleft is not fully understood, there is geographical and race variation, and 30 % of those born with a cleft have other anomalies and are classed as syndromic.^1^ Survival is lower when CLP is associated with other congenital anomalies or syndromes compared to isolated CLP.^2^ There are also associations with environmental exposures such as maternal smoking, alcohol intake, diabetes, and body mass index.^3^ There is clearly a genetic component in causation and there appear to be separate genetic pathways to the different phenotypes.^4^ Clefting is a Global problem with an affected child being born somewhere in the world every three minutes. Brazil is the world's fifth-largest country by area and the seventh most populous comprising well over 200 million. In Brazil, there are about 4.24/10,000 live births with some form of orofacial cleft^5^ and by comparison in a study of registries in 18 countries, the pooled prevalence was 6.4 CLP per 10,000 births.^2^

The diagnosis of the cleft in utero or at birth is a difficult time for parents and families with associated anxieties that extend for many years as the child with a cleft is treated for various cleft-related difficulties. Psychologists have reported extensively on these issues over many years but the requirements for support are still not clear and require more research involving longitudinal cohort studies and an effort to incorporate the patient perspective.^6^^,^^7^ After the birth of a child with a cleft, specialist nursing is needed for feeding advice and where surgery is planned, direct discussions with surgical teams. Surgery is required to repair the cleft lip, the palate and later bone grafting of the maxillary alveolus to unite major and minor alveolar segments. Orthognathic jaw correction may also be required. As speech develops, assessment and treatment will be closely linked with audiology and ENT services. Dental input provides early preventative advice and orthodontists are involved during dental development and preparation for surgical procedures. Dental restorative input may be required in adulthood. This outlines a core team of disciplines where arguably clinical nurse specialists are the constant throughout the care pathway. There is a burden on the child born with a cleft and their family as well as the health service delivering the care. This however has to recognise the wider support needed in caring for these children such as clinical geneticists.

In this issue of *Jornal de Pediatria*, the paper by Silva et al.^5^ provides an informed view as to why genetics needs to be fully integrated into clinical services. It also highlights the need to value and use national registries which is where the data were collected/verified for a defined 10-year period together with a parallel research effort (Brazil's Craniofacial Project, BCFP). The work highlights that there is uneven access to genetic evaluation and diagnostic testing for OC among its participant centers. There is a need to improve the accessibility of genetic testing, particularly within the public health system. It also demonstrates the importance of merging research with clinical services.

How then will care for these children improve? In Brazil, there is recognition that children born with a cleft require integrated and coordinated care within the public health system. Moreover, since 2003 there has been a National Policy for Comprehensive Care for People with Rare Diseases (PNAIPDR) with this clear framework. Financial constraints inevitably limit implementation but the strategy is to be applauded and few other countries are able to achieve the intended aims. Certainly, private healthcare systems will struggle to integrate and co-ordinate care in this way let alone provide any meaningful research. The drive to establish the BCFP came from the PNAIPDR framework and importantly is a direction of how care should ideally integrate all parts. It is now well recognised that dispersed care with fragmented services yields poor outcomes committing these children born with a cleft to potentially tortured lives in society.^8^^,^^9^ There are a few examples of how re-configuring care to a centralized model can improve outcomes^10^ and importantly start to inculcate a research culture embedded in clinical care with early career health carers heavily involved.^11^^,^^12^ In the United Kingdom there has been much progress in cleft care over the last two and half decades since the government directed a centralized service should be developed as well as a national registry[CSAG]. The current configuration comprises 11 managed clinical networks which all complete birth registrations with the national registry.^13^ This centralized service provided an opportunity to establish a research cohort study (Cleft Collective, funded by The Scar Free Foundation, Underwood Trust, Vocational Training Charitable Trust, Medical Research Council, Wellcome Trust, and a number of other charities) which all cleft centers contribute to.^14^ The Cleft Collective is unique, it is a longitudinal cohort study collecting much information from children born with cleft and their families. This includes child, parental, and sibling DNA as well as cord blood where there is ante-natal diagnosis. The Cleft Collective relies heavily on the goodwill of the clinical teams for recruitment and collection of samples. Some of the clinical teams access and publish data, there is some collaboration with CRANE and some connection with the clinical geneticists but optimisation would merge these activities into a seamless service. Silva et al.^5^ make an excellent case for this.

There are few studies that look at the overall cost of a service that integrates all aspects of cleft care. Micro cost studies tend to consider a specific aspect of cleft care rather than a total cost including aspects such as genetics, sonography, imaging, specialist nursing and others. Public health services need to consider this aspect in budgeting and wider studies are needed.^15^

The national registry in Brazil is a strong foundation for establishing an ideal service for children born with cleft. By the size of the population this registry will yield much information and rapidly. By contrast, there are some excellent detailed European registries that have been established for many years but the small populations constrain information.^16^ Sweden has an excellent registry with a high level of coverage and reporting of reliable variables together with longitudinal collection. Regular review of variables provides continuous improvements and modifications. Importantly, variables are realistic, valid, and relevant. The Swedish registry and only two other registries in the world publish their results annually.^17^ The challenge to all national registries is to build on the excellent Swedish model. Silva et al.^5^ point out that integrating access to genetic services is crucial in streamlining genetic counseling and for early identification of syndromic CLP cases that need consideration of other issues. Late diagnosis of syndromes can cause a delay with appropriate interventions.

Information on how genetics and clinical care influence outcomes may be a mechanism for understanding and determine individual care pathways. This could be true for psychology, surgery speech and language therapy, educational support as well as many other areas. Importantly this could improve the care of those children born with a cleft. The need for global collaboration to systematically address the integration of care for children born with cleft and improve long-term outcomes and lives is made clear by Silva et al.^5^

## Conflicts of interest

The author declares no conflicts of interest.
